# Epidemiologic Aspects of an Emerging Focus of Visceral Leishmaniasis in Tbilisi, Georgia

**DOI:** 10.1371/journal.pntd.0001415

**Published:** 2011-12-13

**Authors:** Ekaterina Giorgobiani, Nazibrola Chitadze, Gvantsa Chanturya, Marina Grdzelidze, Ryan C. Jochim, Anna Machablishvili, Tsiuri Tushishvili, Yulia Zedginidze, Marina K. Manjgaladze, Nino Iashvili, Manana P. Makharadze, Tsiuri Zakaraya, Konstantin Kikaleishvili, Ivan Markhvashvili, Goderdzi Badashvili, Teymuraz Daraselia, Michael P. Fay, Shaden Kamhawi, David Sacks

**Affiliations:** 1 National Center for Disease Control and Public Health, Tbilisi, Georgia; 2 Laboratory of Malaria and Vector Research, National Institute of Allergy and Infectious Diseases, National Institutes of Health, Bethesda, Maryland, United States of America; 3 Research Institute of Medical Parasitology and Tropical Medicine, Tbilisi, Georgia; 4 Biostatistics Research Branch, National Institute of Allergy and Infectious Diseases, National Institutes of Health, Bethesda, Maryland, United States of America; 5 Laboratory of Parasitic Diseases, National Institute of Allergy and Infectious Diseases, National Institutes of Health, Bethesda, Maryland, United States of America; University of Pittsburgh, United States of America

## Abstract

**Background:**

Over the last 15 years, visceral leishmaniasis (VL) has emerged as a public health concern in Tbilisi, the capital of Georgia.

**Methodology/Principal Findings:**

Seroepidemiological surveys were conducted to determine the prevalence and incidence of infection in children and dogs within the main focus of VL, and to identify risk factors associated with human infection. Of 4,250 children investigated, 7.3% were positive by direct agglutination test in a baseline survey; an apparent incidence rate of 6.0% was estimated by one year follow-up. None of the seropositive children progressed to VL during the survey. Increased seropositivity at one year was predicted by presence at baseline of clustered flying insects (*OR* = 1.49; *P* = 0.001), perceived satisfactory sanitation (*OR* = 1.65; *P*<0.001), stray dogs (*OR* = 1.33; *P* = 0.023), and by persistent fever during the 6 months prior to baseline survey (*OR* = 14.2; *P*<0.001). Overall, 18.2% (107/588) of domestic and 15.3% (110/718) of stray dogs were seropositive by the rk39 dipstick test. Clinical VL signs were found in 1.3% of domestic and 2.9% of stray, seropositive dogs. Parasites isolated from human and dog samples were identified by PCR and phylogenetic analysis of the *Leishmania* 70 kDa heat-shock protein (*HSP70*) gene as *Leishmania infantum*.

**Conclusions/Significance:**

There is an active focus of *L. infantum* transmission in Tbilisi with a high prevalence of human and canine infections.

## Author Summary

Visceral leishmaniasis (VL) has emerged as a public health problem in Tbilisi, the capital of Georgia. Dogs are the main infection reservoirs for transmission by sand flies of *Leishmania infantum* to humans, many of whom may become infected without developing disease. Since majority of cases are in children we were interested to know the rate of infection in children and in dogs living within the area where cases of VL have been found, and what factors may affect the risk of infection. Using a test that detects the presence of antibodies in blood as a marker of infection, 7.3% of 4,250 children examined were positive at the baseline survey, and 6% became positive after one year. Overall, 18.2% of domestic and 15.3% of stray dogs were seropositive. The infected children were more apt to live in areas where clustered flying insects and stray dogs were observed, and were far more likely to have experienced a persistent fever in the 6 months before the survey. We conclude that there is very active transmission of *L. infantum* to both humans and dogs in Tbilisi, and that children remain at high risk of developing clinical disease and sub-clinical infection.

## Introduction

Visceral leishmaniasis (VL) caused by parasites of the *Leishmania donovani* complex (*L. donovani*, *L. infantum/L. chagasi*) is a severe disease with a fatal outcome if left untreated. It is clinically characterized by low-grade fever, enlarged spleen and liver, and weight loss. Anthroponotic VL caused by *L. donovani* is endemic in East African countries, Northeast India, Nepal, and Bangladesh [1], [2]. In Latin America VL is zoonotic and the causative agent is *L. chagasi* [3]. In the Mediterranean basin zoonotic VL is caused by *L. infantum*, with incidence ranging from 0.02/100,000 to 8.53/100,000 [4]. Dogs are recognized as the primary reservoirs of zoonotic VL [5] and the prevalence of canine VL in Mediterranean countries varies from 1.1% to 48.4% [4], [6].

**Figure 1:**
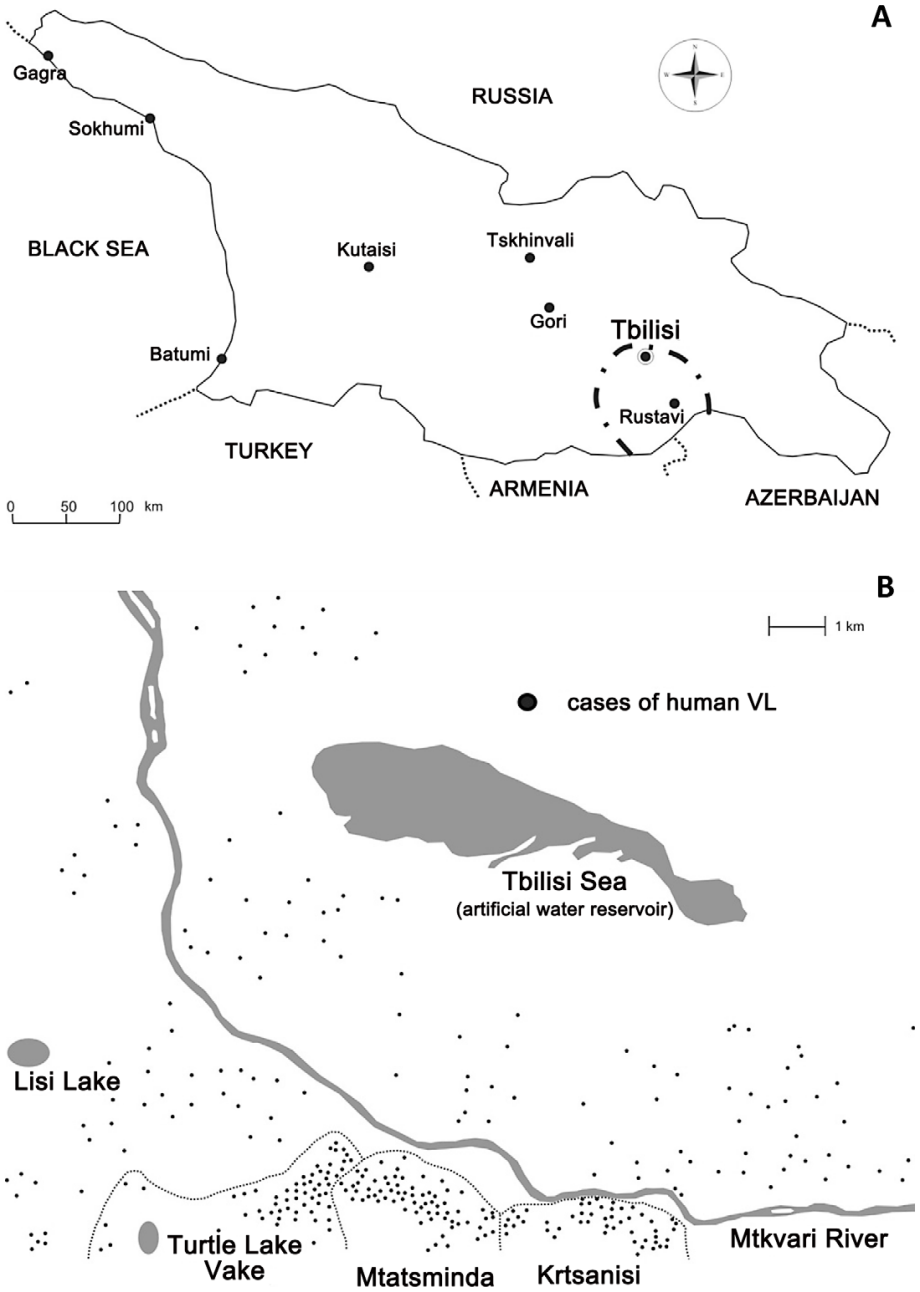
Maps showing distribution of human cases of visceral leishmaniasis in Georgia. Map of Georgia (A) showing the principal active foci of VL in Tbilisi and Shida Kartli region (outlined by —·—). Map of Tbilisi (B) showing distribution of VL cases and location of Krtsanisi, Mtatsminda, and Vake districts surveyed in this study.

Zoonotic VL is widely distributed in countries of the former Soviet Union. Human cases are registered in Middle Asia and the Caucasus, including the Republic of Georgia [7]. Historically, leishmaniasis in Georgia has been sporadic and confined mainly to the eastern part of the country [8]. Current active foci are located in Tbilisi, the capital of Georgia, and in the region Shida Kartli (Figure 1A) [9], [10]. Since 1990, the number of VL cases recorded annually has increased substantially, from 10–12 cases in the early 90's, to 171 cases in 2008. Out of 1535 patients registered in Georgia during 1995–2008, 917 (60%) were from Tbilisi, with 17 fatal cases (official statistical records, National Center for Disease Control (NCDC), Tbilisi, Georgia).

Due to the lack of VL surveillance system in Georgia, the prevalence and incidence of human and canine infection with *Leishmania* has remained unknown. Consequently, an appropriate control strategy has not been formulated. In this paper we present the results of a 3-year prospective study carried out during 2006–2008 in Tbilisi with the aims to determine the prevalence and incidence of *Leishmania* infection in children, as well as its prevalence in domestic and stray dogs, and to confirm the identity of the *Leishmania* parasite responsible for the disease.

## Materials and Methods

### Study area

Tbilisi is located at 500–800 m above sea level and is divided into northern and southern parts by the Mtkvari River. The southern part is mostly hilly with canyons and ravines; the northern part is terraced. Of the 10 urban districts, most of the VL cases (∼70%) registered during 1997–2004 originated from Krtsanisi, Mtatsminda and Vake located in the southern part of the city along the foot of the Mtatsminda Mountain. These 3 districts, representing active foci of VL, were selected as study sites (Figure 1B). The populations of the districts were: Krtsanisi – 27,047 (5,155 children 1–14 yrs of age); Mtatsminda – 43,133 (8,914); Vake – 27,053 (6,855).

### Study design and sample collection

#### a. Human

Since approximately 70% of VL cases have been registered in children less than 14 years old, children 1–14 years of age were selected by randomized probability-proportional-to-size cluster sampling using 2002 census areas as clusters. All 1–14 years old children in each household (n = 4250) were surveyed at baseline and one year follow-up. The survey evaluated the medical history of each child, demographic characteristics of the household, and possible risk-factors associated with infection. After written consent from their parents or guardians, children underwent a physical examination for detection of clinical symptoms consistent with VL. A finger-prick blood sample was collected on filter paper (Whatman No. 3). Parents or guardians of serologically positive children were warned about possible development of the disease and asked to notify NCDC in the event clinical signs of VL occurred. The protocols of the study were approved by the Ethics Committee of NCDC.

#### b. Dogs

A cross-sectional study of domestic and stray dogs was performed to determine seroprevalence rate in canine populations. A total of 588 domestic dogs were selected from households in the human study areas and 718 stray dogs were also chosen from the same streets and neighboring suburbs. Once examined and sampled, a collar was placed on each stray dog to avoid repeated involvement of the same dogs in the survey. Owners provided consent for sample collection involving domestic dogs. Prior to sample collection, all dogs (domestic and stray) were examined for clinical signs of canine VL (alopecia, local or generalized lymphadenopathy, ocular and periocular lesions, epistaxis, onychogrypyhosis, emaciation, splenomegaly). Dogs were anesthetized intramuscularly with 2% acepromazine. Peripheral blood was taken from a femur or foreleg vein in Eppendorf® tubes (≥2 ml) and centrifuged for serum collection. Bone marrow aspirates were obtained from the iliac crest only from seropositive domestic dogs and used for smears, parasite culture and PCR. This animal protocol was approved by the Ethics Committee of the National Center for Disease Control (N05/04-02-492) according to and in compliance with the Georgian legislation and international norms in biological research.

### Parasitological diagnosis

Bone marrow aspirates were smeared onto microscopic slides, dried, fixed with methanol, stained with Giemsa, and examined microscopically under a 100× oil immersion objective for presence of *Leishmania* amastigotes.

### Direct Agglutination Test (DAT)

Standard procedures for human serodiagnosis by DAT [11], [12] were performed using the freeze-dried *Leishmania* antigen (Royal Tropical Institute, Amsterdam, Netherlands) to measure the antibody titer in finger-prick blood. Briefly, blood samples were eluted from filter papers overnight in serum diluent and serially diluted in twofold dilutions from 1∶100 to 1∶51,200 in 96-well plates. The DAT antigen was added to each dilution and the results were read after 18 h of incubation at room temperature.

To determine the cut off point for the DAT, blood samples from 100 children of the same age group living in the non-endemic Adjara region, in the city of Batumi (western Georgia) were tested at dilutions ranging from 1∶200 to 1∶25,600. None of the Batumi samples was positive at or above 1∶6,400 dilutions, thus samples giving titers of ≥1∶6,400 were considered seropositive. This cutoff is higher than is typically used for serodiagnosis of active VL [11], [12], therefore the specificity of the DAT in our survey should be at least as high as seen in the previous studies (119/124 = 96% in a mostly endemic population [11], 434/435 = 99.8% in a population with various other diseases [12]). Using the 1∶6,400 cutoff for detection of active cases in an endemic population, Oskam et al. [13] observed perfect specificity of the DAT (130/130 = 100%).

Samples that produced borderline agglutination at ≥1∶6,400 were scored as suspected positive, although the prevalence and incidence rates were calculated from only ‘confirmed’ seropositives. The suspected positives were included in the denominators of these calculations. Blood samples from patients with confirmed diagnosis of VL provided by the Research Institute of Medical Parasitology and Tropical Medicine (RIMPTM, Tbilisi, Georgia) were used as positive controls, and blood samples from DAT negative healthy individuals without signs or symptoms of leishmaniasis were used as negative controls.

### rK39 dipstick test

The Kalazar Detect rapid test was performed according to the manufacturer's protocol (InBios International, Inc. Seattle, WA, USA) using 20 µl for each canine serum sample. The test was considered positive when a control line and test line appeared in the test area within 10 minutes, negative if only a single control line appeared, and invalid if none of the lines appeared.

### *Leishmania* species identification

PCR analysis was performed to identify *Leishmania* parasites isolated from dogs and humans. DNA was extracted using the DNeasy Blood & Tissue Mini Kit (QIAGEN) from bone marrow cultures isolated from serologically positive domestic dogs and a culture established from the bone marrow of a patient with clinically confirmed VL diagnosis at the RIMPTM.

The primers Uni21 (5′ GGG GTT GGT GTA AAA TAG GCC 3′) and Lmj4 (5′ CTA GTT TCC CGC TCC GAG 3′) were used for initial PCR analysis [14]. The PCR was performed in a 25 µl reaction mixture, containing 12.5 µl of 2× FastStart PCR Master (Roche), 1 µl of each primer (10 µM), and 5 µl of template DNA (∼20 ng). Conditions for the reaction were as follows: initial denaturation at 94°C for 5 min, amplification for 35 cycles at 94°C–1 min, 60°C–1 min, 72°C–1.5 min, elongation at 72°C–10 min, and holding at 4°C. The PCR products were separated in 1.4% agarose gel, stained with ethidium bromide and visualized by ultraviolet light. *L. infantum* (MHOM/ES/00/UCM-1) and *L. major* MHOM/IL/80/Friedlin reference strains were included for comparison.

Further *Leishmania* identification was achieved by amplification of the 70 kDa heat-shock protein (*HSP70*) gene from dog and human samples using primers HSP70sen (5′-GACGGTGCCTGCCTACTTCAA-3′) and HSP70ant (5′-CCGCCCATGCTCTGGTACATC-3′) [15]. The PCR was completed in a volume of 50 µl contained 5 µl of 10× PCR Buffer II, 1 µl of each primer (10 µM), 50 ng of template DNA, 1 µl of *Taq* DNA polymerase (AccuPrime™ *Taq* DNA Polymerase System; Invitrogen) and 16 µl of H_2_O. The samples were incubated at 94°C for 2 minutes followed by 35 cycles of 94°C for 30 seconds, 60°C for 30 seconds and 68°C for 1.5 minutes. The amplified 1422 bp products were separated by electrophoresis through 1.2% agarose gel and visualized using SYBR Safe DNA gel stain (Invitrogen). The excised bands were extracted with Ultrafree-DA DNA Extraction (Millipore) and cleaned with three washes of ultrapure H_2_O through an YM-30 Microcon filter (Millipore). The cleaned products were ligated into pCR 4-TOPO (Invitrogen) and the plasmid transformed into One Shot TOP10 competent *Escherichia coli* (Invitrogen). Colonies were screened by PCR with HSP70sen and HSP70ant primers. Positive colonies were grown overnight and the plasmids purified with the PureLink Quick Plasmid Miniprep Kit (Invitrogen). The plasmids were sequenced bidirectionally with M13R (5′-CAGGAAACAGCTATGACC-3′) and M13F (5′-TGTAAAACGACGGCCAGT-3′).

Nucleotide sequences of the *HSP70* gene of *Leishmania* parasites from dog and human samples were aligned against published sequences for *L. infantum*, *L. donovani*, *L. tropica*, *L. major* and *L. aethiopica* strains [16] using Clustal X, version 1.83 [17]. Phylogenetic analysis was conducted on the alignments using MEGA, version 3.1, generating trees by neighbor-joining and testing phylogeny with 1,000 replications to calculate node support [18].

### Statistical analysis

Prevalence and incidence rates with confidence intervals were estimated using logistic models with generalized estimating equations (GEE) with a working independence assumption [19] to account for the within-family correlation. The responses for the logistic models were either the baseline DAT tests (for prevalence) or the one-year DAT tests only from those who were negative at baseline (for incidence). These estimates make no adjustment for the fact that the DAT is an imperfect diagnostic tool. However, because we expect the specificity to be near 100% (see DAT section above), and because imperfect sensitivity will only cause the apparent prevalence to be higher than the true prevalence [20], our prevalence estimates should be interpreted as estimating the lower bounds on the true prevalence. Our incidence estimates should also be interpreted cautiously as apparent incidences, since the adjustments needed to estimate true incidence [20] require sensitivity estimates. While the DAT sensitivity studies in the literature [21] typically refer to clinical disease for which the DAT results can be readily validated by parasite detection, our interest was subclinical infection for which there is so far no proven way to confirm the presence of parasites in the blood or other tissues.

We predicted seropositivity after one year using baseline disease status, region, and answers to survey questions at baseline using GEE cumulative logit models on the ordered responses: negative, suspected positive, and confirmed positive. We eliminated several survey questions that essentially duplicated other questions, and with the remaining questions we built one overall GEE model. Additionally, we built separate GEE models for each survey question using only that question plus baseline disease status and region. Odds ratios (ORs) are ratios of either the odds of confirmed seropositivity or the odds of suspected seropositivity [22]. ORs for yes/no questions estimate the ratio of the odds of disease for a subject who answered “yes” over the odds for a different hypothetical subject who answered “no” but is matched on all other variables in the model. Analyses were done in SAS version 9.1 with a p-value of <0.05 considered statistically significant.

## Results

### Seroprevalence and incidence of VL in children

We obtained baseline DAT responses on 99.6% of the subjects for a total of 4,250 children from 2,968 households; 1,459 children (34%) were 1–4 years and 2,791 (66%) were 5–14 years; 2,096 (49%) were girls and 2,150 (51%) were boys. The overall baseline prevalence was 7.3% with an incidence rate, estimated by a one year follow-up study, of 6.0%. The baseline seropositivity was highest in Mtatsminda (10.6%) followed by Vake (6.3%) and Krtsanisi (0.5%), while the incidence rate was highest in Vake (7.9%) followed by Mtatsminda (5.2%) and Krtasanisi (4.5%) (Table 1).

**Table 1:** Baseline prevalence and annual incidence (one year follow-up) of *Leishmania* seropositivity in surveyed children.

Study area	Prevalence^a^		Incidence	
	N positive at baseline/N total at baseline (%)	95% CI^b^	N positive at follow up and negative at baseline/N negative at baseline (%)	95% CI^b^
Vake	95/1520 (6.25)	(5.06–7.70)	110/1388 (7.93)	(6.26–9.99)
Mtatsminda	211/1995 (10.58)	(9.17–12.17)	92/1777 (5.18)	(4.02–6.64)
Krtsanisi	4/735 (0.54)	(0.21–1.44)	33/731 (4.51)	(3.11–6.52)
Total (all districts)	310/4250 (7.29)	(6.48–8.20)	235/3896 (6.03)	(5.15–7.05)

a Prevalence estimates do not include two children with active form of VL detected during the study.

b 95% confidence interval calculated using GEE methods to account for within family correlation.

**Figure 2:**
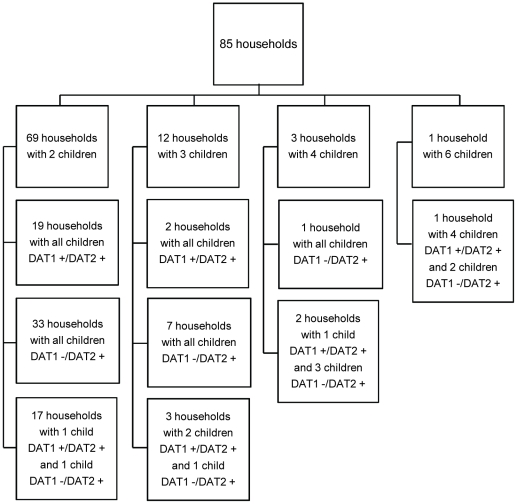
Households with all children tested seropositive for *Leishmania* at the baseline and/or follow-up surveys. Eighty five households with 2 or more children where all children were DAT positive either in both baseline and follow up surveys, or seroconverted during the follow up period: DAT1− children negative in baseline survey, DAT1+ children positive in baseline survey, DAT2+ children positive in follow-up survey.

None of the seropositive children who were asymptomatic at baseline developed clinical signs of VL during the follow-up period, although 7% (296 children) of all investigated children were seropositive during both the baseline survey and one year follow-up. Fourteen of 310 children (4.5%) who were positive at baseline became negative in the follow-up survey. In 85 households with 2 or more children, all children were positive either in both baseline and follow up surveys, or seroconverted during the follow up period (Figure 2).

Hepatosplenomegaly, jointly with other clinical signs of active disease (weight loss, fatigue, anorexia, fever of unknown etiology), was detected in 2 children during clinical examination in the baseline survey. These children were excluded from the study and sent to the RIMPTM where the diagnosis of VL was parasitologically confirmed. Twenty two children with a previous history of VL were identified during the baseline study. Of these children, 11 (50%) were positive by DAT both in the baseline and follow-up surveys.

We predicted seropositivity at follow-up from only baseline disease status, region, and each baseline variable alone (Table 2, unadjusted OR) or by a complete model with all baseline variables included (Table 2, adjusted OR). We found that by both unadjusted and adjusted methods the odds of seropositivity at follow-up were increased by clustered flying insects at sunset/sunrise, perceived satisfactory sanitary conditions, and stray dogs; odds were decreased by the presence of nearby woodlands. The leading risk factor positively associated with seropositivity, with an *OR* of 14.2 (unadjusted) or 13.6 (adjusted) (*p*<0.001 for both methods), was fever lasting more than 2 weeks for which antibiotic therapy was not effective, and occurring during the 6 months prior to the day of the baseline interview (Table 2). No association of seropositivity by either method was found in relation to age or gender, or the use of nets on doors and windows. The use of repellents and the nearby existence of facilities for domestic animals were significant only after adjusting for the other variables. A history of lack of appetite at baseline was predictive of disease one year later but this effect disappears after adjusting for the other variables. Importantly, there was no association of seropositivity with having a pet dog(s) at home or in the yard by either method (Table 2).

**Table 2:** Odds ratios from unadjusted and adjusted cumulative logistic models^a^ predicting seropositivity of children at follow-up.

Parameters	Unadjusted OR^b^ (95% CI)	P value	Adjusted OR^c^ (95% CI)	P value
Fever lasting more than 2 weeks during the last 6 months for which antibiotic therapy was not effective	14.2 (6.1–32.7)	<0.001	13.6 (4.5–40.7)	<0.001
Woodlands with deciduous trees	0.50 (0.38–0.67)	<0.001	0.66 (0.46–0.95)	0.024
Perceived satisfactory sanitary conditions	1.65 (1.28–2.14)	<0.001	1.68 (1.25–2.28)	<0.001
Clustered flying insects during the sunset/sunrise	1.49(1.17–1.90)	0.001	1.58 (1.16–2.15)	0.004
Stray dogs	1.33 (1.04–1.70)	0.023	1.72 (1.28–2.32)	<0.001
Anorexia and/or sudden weight loss during the last 6 months	8.2 (2.4–28.6)	0.001	0.89 (0.18–4.38)	0.888
Pet dog at home	1.30 (0.75–2.26)	0.349	1.24 (0.63–2.44)	0.525
Dog in the yard	0.50 (0.24–1.02)	0.056	0.35 (0.11–1.06)	0.062
Facilities for poultry or animals in the yard or neighboring area	0.69 (0.42–1.12)	0.133	0.50 (0.26–0.96)	0.036
Rodents or their burrows in nearby area	0.99 (0.79–1.24)	0.929	0.83 (0.62–1.11)	0.208
Use of repellents for protection from insects	0.86 (0.65–1.14)	0.304	0.66 (0.47–0.94)	0.020
Use of nets on doors and windows	1.02 (0.82–1.28)	0.843	1.00 (0.78–1.30)	0.993

a Logistic models with generalized estimating equations (GEE) were used to predict disease status in 1–14 year old children in one year after baseline survey.

b Model includes only baseline disease status and region plus the variable listed in the first row.

c Model includes variable listed in first column plus: baseline disease status, previous diagnosis of Leishmaniasis, whether children visit parks in the evening, region, age (0–4, 5–9, 10+), sex, type of dwelling (isolated apartment, municipal apartment, private house), and floor.

### Seroprevalence of VL in dogs

Overall, 107 of 588 domestic dogs (18.2%) and 110 of 718 stray dogs (15.3%) were found seropositive by the rK39 dipstick test (Table 3). Among the domestic dogs surveyed, the highest seroprevalence rate was in Vake – 31.2% (81 of 260 dogs), followed by Krtsanisi – 10.1% (14 of 138), and Mtatsminda – 6.3% (12 of 190). In the stray population, however, the highest seroprevalence rate was observed in Krtsanisi – 19.9% (36 of 181), followed by 14.5% (48 of 331) in Vake, and 12.6% (26 of 206) in Mtatsminda (Table 3). Clinical signs of canine VL were found only in 1.9% (2 of 107) and 2.7% (3 of 110) of seropositive domestic and stray dogs, respectively. However, the presence of *Leishmania* amastigotes was confirmed by microscopy in 49 of 75 (65%) bone marrow aspirates taken from seropositive domestic dogs. No statistically significant difference was observed between seropositivity and gender or age of investigated dogs. A difference in seroprevalence according to breed was observed among the 33 breeds and mongrels included in this study. A higher seropositivity was observed in Doberman Pinchers – 3 of 3, hounds – 4 of 5, pit bulls – 7 of 16, European shepherds – 2 of 6, German shepherds – 23 of 70, Drathaars – 1 of 3; compared to Caucasus shepherds – 1 of 31, Rottweilers – 1 of 15, poodles – 3 of 31, and mongrels – 31 of 232.

**Table 3:** Prevalence of *Leishmania* seropositivity in domestic and stray dogs from the study area.

Study area	Domestic dogs		Stray dogs	
	N positive/N total (%)	95% CI	N positive/N total (%)	95% CI
Vake	81/260 (31.2)	(25.6–37.2)	48/331 (14.5)	(10.9–18.8)
Mtatsminda	12/190 (6.3)	(3.3–10.8)	26/206 (12.6)	(8.4–17.9)
Krtsanisi	14/138 (10.1)	(5.7–16.4)	36/181 (19.9)	(14.3–26.5)
Total (all districts)	107/588 (18.2)	(15.2–21.6)	110/718 (15.3)	(12.8–18.2)

### *Leishmania* species identification

**Figure 3:**
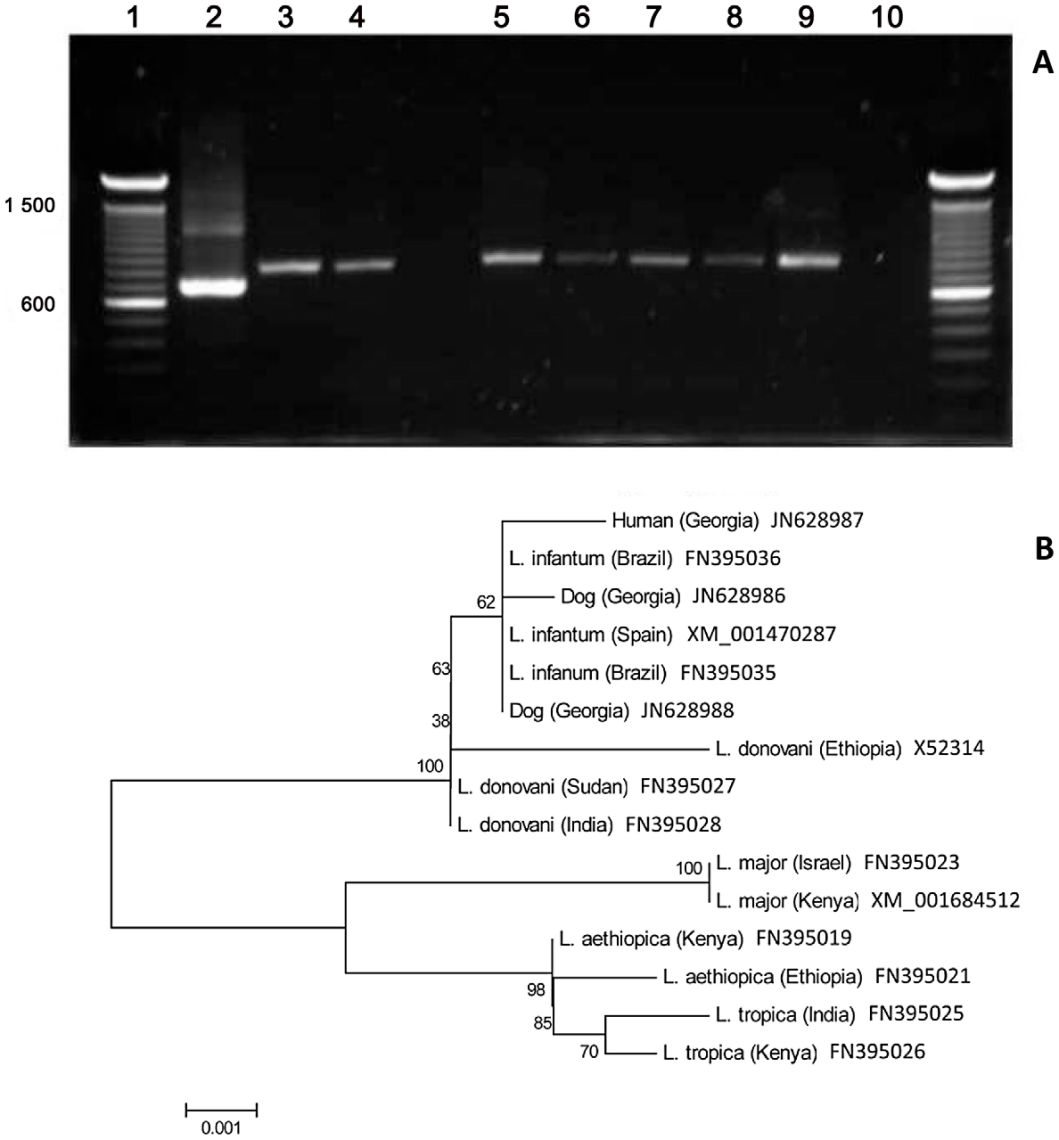
Identification of *Leishmania* species isolated from a human and dogs. (A) kDNA-PCR performed on DNA extracted from the bone marrow of a sick child and bone marrow cultures of five representative dogs. Lane 1, DNA size marker (100 bp ladder); lane 2, *L. major* (MHOM/IL/80/Friedlin); lane 3, *L. infantum* (MHOM/ES/00/UCM-1); lane 4, bone marrow, child, Tbilisi; lanes 5–9, bone marrow, dogs, Tbilisi; lane 10, negative control. (B) Phylogenetic analysis of *Leishmania* 70 kDa heat-shock protein (*HSP70*) genes. The sequences are represented by the *Leishmania sp.*, country of origin in parentheses and GenBank nucleotide accession number. Node values indicate branch support.

Amplification of DNA extracted from bone marrow of five seropositive dogs and one infected child with parasitologically confirmed VL produced single bands of approximately 800 bp identical in size to the *L. infantum* reference strain (Figure 3A). No amplification was observed in the negative control without template DNA.

Phylogenetic analysis of the 70 kDa heat-shock protein (*HSP70*) nucleotide sequences from DNA extracted from the dog and human bone marrow confirmed the identity of the parasite as *L. infantum* (Figure 3B).

## Discussion

We report results of the first large seroepidemiological study of an important, emerging focus of human and canine VL in Tbilisi. Overall, 310 (7.3%) of 4,250 investigated children were found seropositive by DAT at the baseline survey, with 235 out of 3,896 seronegative children (6.0%) converting to positive within a one year follow-up. The choice of DAT as the serological test was justified by its prior application in a large number of field-based epidemiologic surveys of VL [23]–[25]. Selection of the cut-off titer of ≥1∶6,400 was based on results obtained from an extensive survey of children living in a non-endemic region of Georgia. For comparison, DAT titers of ≥1∶3,200 have been used by different investigators as a cut-off for the assessment of *L. donovani/L. infantum* seropositivity [23], [25]–[28], although Harith et al. [23] suggested a single serum dilution of 1∶6,400 for use in mass screenings. Moreover, they compared filter paper eluates with the corresponding serum samples and found no significant difference in DAT titers [23].

The true prevalence is likely higher than 7.3% because of the imperfect sensitivity of the DAT at the 1∶6,400 cut-off. With imperfect sensitivity, some of those that have VL will not be detected. By using probability laws, and assuming the sensitivity and specificity are known, we can get an estimate of the true prevalence [20] (see Text S1): True Prevalence = (Apparent Prevalence+Specificity−1)/(Sensitivity+Specificity−1). Using data on clinical VL from Oskam et al. (1999), we estimated the specificity as 100% (130/130) and the sensitivity as 71.7% (147/205), so that the corrected estimate of true prevalence using the above formula is 10.2%. These calculations depend on the estimates of sensitivity and specificity, and if the specificity is less than 100% then the True Prevalence estimate would lower than 10.2%, while if the sensitivity is lower (which we might expect for detecting subclinical disease) then the estimated true prevalence would be higher than 10.2%.

Different prevalence rates of asymptomatic seropositive humans have been found in Greece (0.5%) [6], Turkey (2.6%) [29], France (10%–28%) [30], Israel (3.0%) [31], Iran (1.6%) [32], and Azerbaijan (8.0%) (neighboring country of Georgia) [33]. Our data indicate the existence of a high frequency of asymptomatic human carriers in Tbilisi that conforms with results of studies reporting circulation of *L. infantum* in peripheral blood of asymptomatic healthy individuals [34]–[36]. All of the 310 children who were seropositive at baseline remained asymptomatic during the one year follow-up, and only 14 (4.5%) reverted to seronegative. Reversion of seropositive asymptomatic individuals to seronegative is well described [37], for example, in Kenya, where 75% of seropositive asymptomatic individuals became seronegative within 12–36 months without developing clinical signs of VL [38], or in Spain, where only 50% of the *L. infantum* asymptomatic seropositive blood donors remained positive after one year [39]. The question of how such changes in serological titers reflect changes in parasitemia remains unresolved.

Interestingly, we found clusters of seropositive children in 85 households with more than one child where all children living in the same household were either positive for anti-*Leishmania* antibody in both baseline and follow-up surveys, or converted to positive in the follow-up survey. None of these children developed clinical signs of VL during our study. Of these households, 33 (39%) were located in Vake district and half of them (17 households) on the same street; 48 households (56%) were in Mtatsminda district, and only 4 households (5%) in Krtsanisi district. Existence of these clusters, along with the absence of an association of seropositivity with domestic dogs, is consistent with the possibility that asymptomatic human cases serve as infection reservoirs.

One important objective of our survey was identification of the potential risk factors associated with seropositivity. We found that children who had persistent fever within the 6 months prior to the interview had a much higher probability (*OR* = 14.2 unadjusted; *p*<0.001) of becoming seropositive than children without this symptom. Some investigators have classified seropositive patients without any symptoms of VL as asymptomatic and seropositive patients having one or a combination of mild symptoms as subclinical. Subclinical patients can progress to the classic form of VL or resolve their symptoms over different periods of time [32], [37], [40], [41]. The seropositive children in our study with no other symptoms but fever can be considered as having a subclinical form of VL, and represent a significant and under reported morbid condition associated with *L. infantum* infection in this population.

Children living in the households that were perceived to have unsatisfactory sanitary conditions were at significantly lower risk of infection (*p*<0.001, both adjusted and unadjusted), as this perception may be associated with more attention to sanitary issues. Children living where clusters of blood-sucking insects were reported had higher risk of infection (*p* = 0.001 unadjusted, p = 0.004 adjusted). The results of the analysis indicated no significant association of seropositivity with sex or age of the investigated population, which conforms to the data from similar studies [6], [42], [43]. The presence of domestic livestock in the yard or the use of screens on the doors and windows or repellents against insects also did not have a statistically significant association with seropositive children. Decreased association with seropositivity in the presence of woodlands (*p*<0.001, unadjusted; *p* = 0.024, adjusted) suggests that woodlands act as a barrier to sand fly-human contact, likely due to the presence of wild animals as a preferred blood meal source.

Although Gavgani et al. [44] suggested that domestic dogs represent a significant risk factor for human VL, our findings did not show any association of seropositive children with domestic dogs, similar to other studies [35], [43]. By contrast, our analysis showed significant correlation with the nearby presence of stray dogs (, *p* = 0.024, unadjusted; *p*<0.001, adjusted). The increased risk of infection is associated with a high overall seroprevalence rate in our survey detected by the rK39 dipstick test for stray dogs (15.3%). This conforms well with the range of average seroprevalence rates of canine leishmaniasis reported from different Mediterranean countries: 20% in Portugal, 8.5% in Spain, 4–20% in France, 2–15% in Italy, 25% in Greece, 20% in Cyprus, 15.7% in Turkey [4]. It is curious that there was no association with seropositivity and the presence of domestic dogs despite the fact that the seroprevalence rate in domestic dogs was even higher (18.2%).

A high prevalence of infected asymptomatic dogs within *L. infantum* endemic areas of Spain, Italy, Brazil, Iran has been a consistent finding [5], [45]–[47]; lower rates were reported from Greece, Azerbaijan and Turkey [6], [33], [48]. While some authors reported that sand flies were not able to acquire infections from asymptomatic dogs [5], others proved that transmission to the vector from asymptomatic, seropositive dogs was possible [49]–[51]. Importantly, our own studies revealed that 49 of 75 bone marrow aspirates taken from seropositive domestic dogs were positive for amastigotes by microscopy, confirming their potential as infection reservoirs.

As for the risk factors associated with dogs becoming seropositive, there was no association with sex or age of dogs, or as mentioned, with domestic versus stray dogs. In contrast to our results, Gavgani et al. [44] showed significantly higher seroprevalence of *L. infantum* in stray (26.6%) in comparison with domestic (12.7%) dogs. Several studies showed no specific correlation between seropositivity and sex of dogs [47], [52], [53], although age-related differences supporting higher rates in older dogs have been reported [6], [47]. The influence of breed of dogs on seroprevalence is also inconclusive [6], [52], [53], though in general, higher rates of canine VL were reported in German shepherds, Dobermans, boxers and hounds, which favorably support the results of our survey.

Numerous PCR-based methods have been used for detection and identification of *Leishmania* parasites, including amplification of the kinetoplast DNA (kDNA) [14], [54]–[58]. Using the Uni21 and Lmj4 primer pair [14], the 800 bp amplicon was obtained from bone marrow DNA of five serologically positive dogs and one child with parasitologically confirmed VL. Though *Leishmania*-specific, this PCR product size is similar in *L. infantum*, *L. donovani* and *L. tropica*. Therefore, the identity of the *Leishmania* species from our study area was confirmed as *L. infantum* by sequence analysis of the 70 kDa heat-shock protein (*HSP70*), demonstrating that this species is responsible for human and canine VL in Tbilisi.

Taken together, our results confirm the presence of an extremely active focus of VL in Tbilisi with a high prevalence of human and canine infections. Importantly, recent data, demonstrating the spread of human VL cases to other parts of Tbilisi and to non-endemic territories of Georgia, indicate an urgent need to formulate and implement effective control measures.

## Supporting Information

Text S1 **Appendix: Derivation of prevalence adjustment formula.**
(DOC)

Checklist S1 **STROBE Checklist.**
(DOC)
