# Supplementary material for: Epidemiologic Aspects of an Emerging Focus of Visceral Leishmaniasis in Tbilisi, Georgia
Source: PLoS Negl Trop Dis. 2011 Dec 13;5(12):e1415. doi: 10.1371/journal.pntd.0001415 (PMC3236723; doi:10.1371/journal.pntd.0001415)
Supplement: Text S1 — Appendix: Derivation of prevalence adjustment formula. (DOC) [file pntd.0001415.s001.doc]

**Text S1. Appendix: Derivation of Prevalence Adjustment Formula**

For completeness we give a derivation of the true prevalence formula of [1]. Let T+ and T- be a positive and negative test, and let D+ and D- be the presence or not of disease. Then sensitivity is the probability of a positive test given you have the disease, or in mathematical notation,

P(T+ | D+). In this notation, specificity is P(T-|D-). Then the probability of observing a positive test is


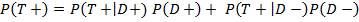


by the theorem of total probabilities [2]. Then replacing P(D-) with 1-P(D+), and P(T+|D-) with 1-P(T-|D-), we get

P(T+) =
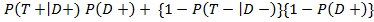
.

Using algebra we write the equation with the true prevalence, P(D+), on the left-hand side as


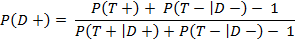


which is the formula given in the text written in mathematical notation.

**References**

1. Yanagawa T, and Gladen BC (1984) Estimating disease rates from a diagnostic test. Am J Epidemiol 119: 1015-1023.

2. Dudewicz, EJ and Mishra, SN (1988) *Modern Mathematical Statistics*. Wiley: New York.
